# Single phase extraction method for determination of dithianon residues in fruits and vegetables using LC ESI (-) MS/MS

**DOI:** 10.1038/s41598-025-23528-4

**Published:** 2025-10-31

**Authors:** Esmail Elsayed Aboshanab, Mahmoud Hamdy Abdelwahed, Sanaa A. M. El-Sawi, Mostafa M. Emara, Ahmed A. Omran

**Affiliations:** 1 Agricultural Research Center (ARC), Central Laboratory of Residue Analysis of Pesticides and Heavy Metals in Food (QCAP Laboratory), Ministry of Agriculture and Land Reclamation, Dokki, Giza, 12311 PO Egypt; 2 Department of Chemistry, Faculty of Science, Al-Azhar University, Nasr City, 11884 Cairo, PO Egypt

**Keywords:** Dithianon, Pesticides residues, LC-MS/MS, Validation, Fruits, Vegetables, Biochemistry, Chemistry, Environmental sciences, Plant sciences

## Abstract

Dithianon is a non-systemic fungicide, applied in some agricultural products. Dithianon residues in food cause health problems for humans so it is recommended to be analyzed in fruits and vegetables. Four different extracting solvents were compared to get the optimum one. Quantitative analysis was done using a liquid chromatography triple quadruple mass spectrometer in different agricultural products. The in-house validation process was carried out based on SANTE guideline. The results demonstrated an average recovery rate between 85 and 113%, with relative standard deviations (RSDs) ≤ 8% for all tested food matrices in repeatability and RSD_wR_% = 16% in within-Laboratory reproducibility. Good linearity at r2 > 0.99 was obtained for 0.001–0.5 µg/ml dithianon calibration curves. Limit of quantifications (LOQs) for the method ranged between 0.01 and 0.05 µg/g with expanded measured uncertainty Uexp = ± 42%. Our method is simple, fast and reliable for the determination of dithianon residues in food so, it is recommended to be applied in the routine analysis. The method’s practicality was confirmed by analyzing fifty market samples from Egypt. No dithianon residues were detected, a finding consistent with its limited national registration and underscoring the method’s utility for ensuring compliance and food safety.

## Introduction

Pesticides refer to chemical substances that are utilized globally to eradicate or manage unwanted plants insects, fungi, and other pests that can damage crops^1,2^. Although pesticides can enhance crop yield, improve their quality, and prolong their shelf life^3,4^, numerous types of pesticides are dangerous to human beings^5–7^ due to their toxic and long-lasting nature^8^.

Dithianon is known as 5,10-Dihydro-5,10-dioxonaphtho(2,3-b)−1,4-dithiine-2,3-dicarbonitrile according to IUPAC^9,10^ which is a wide-spectrum non-systemic fungicide with curative action for some foliar diseases in some agricultural products especially in fruits^11,12^ and vegetables^13^. The presence of fungal diseases such as scab on apples^14,15^, leaf curl of peaches, Diplocarpon earlier on strawberries^16,17^ and Phomopsis citri^18^ on citrus fruits^19^ are involved the application of common fungicides during the plant’s growing^20^ season for example applying of dithianon as a fungicide to control of Blumeriella jaapii and Stigmina carpophila^21^.

According to WHO our target dithianon pesticide has a moderately hazardous (Class II) in its toxicity class^6,22–24^. Due to the toxicity effect of dithianon, a strict legislative framework is established to control the maximum amount permitted in food and protect human health^25–27^. In countries such as the USA, EU, Japan, and the Republic of Korea, the maximum residue limits (MRLs) of dithianon that can be legally detected in/on crops have been established in various crops in the range from 0.01 to 100 µg/g^7,28^. According to the previous studies, there are a few different analytical methods for the determination of dithianon residues in food that were established. In 2012, Tian et al. developed an analytical method for the determination of dithianon in water and fruits by old fashion technique spectrofluorimeter with LOQ equal to 10.6 µg/g which is relatively high^28,29^.

A study conducted by Jin Jang et al. in 2013 to analyze dithianon residues in red pepper, carried out using high-performance liquid chromatography with ultraviolet detector (HPLC-UV) with long time clean-up step^30^, performed using a silica solid-phase extraction (SPE) cartridge^7^.

In 2018, Sanja LAZIC et al. employed the quick, easy, cheap, effective, rugged and safe (QuEChERS) method with clean-up steps to extract dithianon residues by using high-performance liquid chromatography with diode array detector (HPLC-DAD). It was noted that this analytical method is associated with high costs and time-consuming procedures with a relatively high limit of quantification (LOQ) of 0.09 µg/g^21^.

Liquid chromatography^7,31^ coupled with triple mass spectrometer (LC-MS/MS)^32,33^ is a recommended analytical technique for determination of dithianon residues and other pesticide residues^34^ in food due to its capabilities in terms of sensitivity, selectivity and structural elucidation^35,36^.

Based on a previous study, it was found that the extraction of dithianon using QuEChERS^37^ was challenging due to its physicochemical characteristics specifically, its polarity. Dithianon is a neutral mid-polar organic pesticide that was decomposed^38^ and hydrolyzed by the effect^39^ of buffered salts in the QuEChERS^40^ extraction method.

In 2024, a core-shell substrate consisting of AuNr/AgNCs was developed to detect dithianon in river water and apple juice with LOD equal to 20 nM^41^.

In our study, development and optimization of the extraction and detection of dithianon residues were carried out by the direct extraction with Ethyl acetate with 1% acetic acid as extracting solvent to overcome the last extraction problems in fruits such as apple and lemon as well as vegetables such as lettuce and spinach using short run time at 12 min by LC-MS/MS injection method.

Finally, the developed method was validated in different food commodities, according to the EU validation guidelines, SANTE/11,312/2021^42^.

## Materials and methods

### Chemicals and solvents

Dithianon reference standard (98.0%) was supplied from Dr. Ehrenstorfer (Augsburg, Germany). LC-MS analytical grade were used during the extraction and chromatographic analysis (99.9%) Acetonitrile, (99.9%) Methanol, (99.8%) Ethyl acetate, (99%) Formic acid, (99%) Acetic acid, were obtained from Fisher Scientific. Ammonia solution (33–35%) was purchased from Fluka (Riedel-de Häen, Germany). Acetone with purity (99%) obtained from Merck (Darmstadt, Germany). Ultrapure deionized water (DIW) was prepared using Milli-Q water purification system (Merck, Germany). 99% Magnesium Sulfate was obtained from (Techno pharmachem, India), and 99.9% Sodium Chloride was purchased from (Tekkim company). A PTFE (polytetrafluoroethylene) syringe filter. Mobile phase (A) a mixture of DIW: MeOH (9:1, v: v) with a pH of 4, containing ammonium formate as a solvent; and (B) pure MeOH.

### Preparation of dithianon standard solutions

A stock solution of 1000 µg/ml dithianon was prepared as an analytical standard solution in Toluene. Working and spiking standard solutions at concentration 5 µg/ml was prepared by diluting of the stock solution in Toluene. Calibration standard levels at the concentrations of 0.001, 0.002, 0.01, 0.05, 0.1, and 0.5 µg/ml were freshly prepared by a serial dilution of the working solution in Methanol 1% formic acid. Stock solution and spiking solution of dithianon were stored at −20 °C for 1 year while the calibration levels were stored at 4 °C for 1 Month. All standard solutions were stored in dark plastic tubes until analysis.

### Apparatus

Polytetrafluoroethylene (PTFE) membrane syringe filter with 0.45 μm pore size and polypropylene centrifuge falcon tubes (50 and 15 ml) with screw caps from Supelco (Bellefonte, USA), Lab Z32 HK mega-fugue centrifuge from (HERMLE, Germany), Geno/Grinder SPEX^®^ sample preparation shaker device (SPEX, USA), bottle top dispenser (5–50 ml) (Hirschman, Germany), fixed micropipette 50 µl and variable micropipettes (2–20 µl) and (200–1000 µl) (Eppendorf, Germany), bench top pH-meter, analytical balance and precision balance (Mettler-Toledo, Selangor), and Grindomix Knife Mills GM 300 grinding machine (Retsch GmbH, Germany) were supplied^43^.

Water purification system MilliQ UF-Plus system (Millipore, Germany) was purchased. AB SCIEX Triple Quad 6500 + LC/MS/MS system equipped with Ion Drive Turbo V source was used, in negative electrospray ionization (ESI) mode coupled with shimadzu HPLC system (Exion LC) was obtained.

### Sample preparation

Various food commodities from fruits and vegetables, including apples, orange, lettuce, and spinach were collected from local Egyptian markets as organic dithianon free samples, used in performing the method validation. About 500 g from our fresh samples were firstly cut into small pieces by knife, then homogenization of the cutted samples by blenders Grindomix Knife Mills GM 300 to get 300-µm particular size to get homogenized sample, and we take care from the heat effect, produced during the grinding process to avoid degradation of our targeted analyte. The homogenized samples were sampled in plastic bottle until analysis it.

### Extraction protocol

**Fig. 1:**
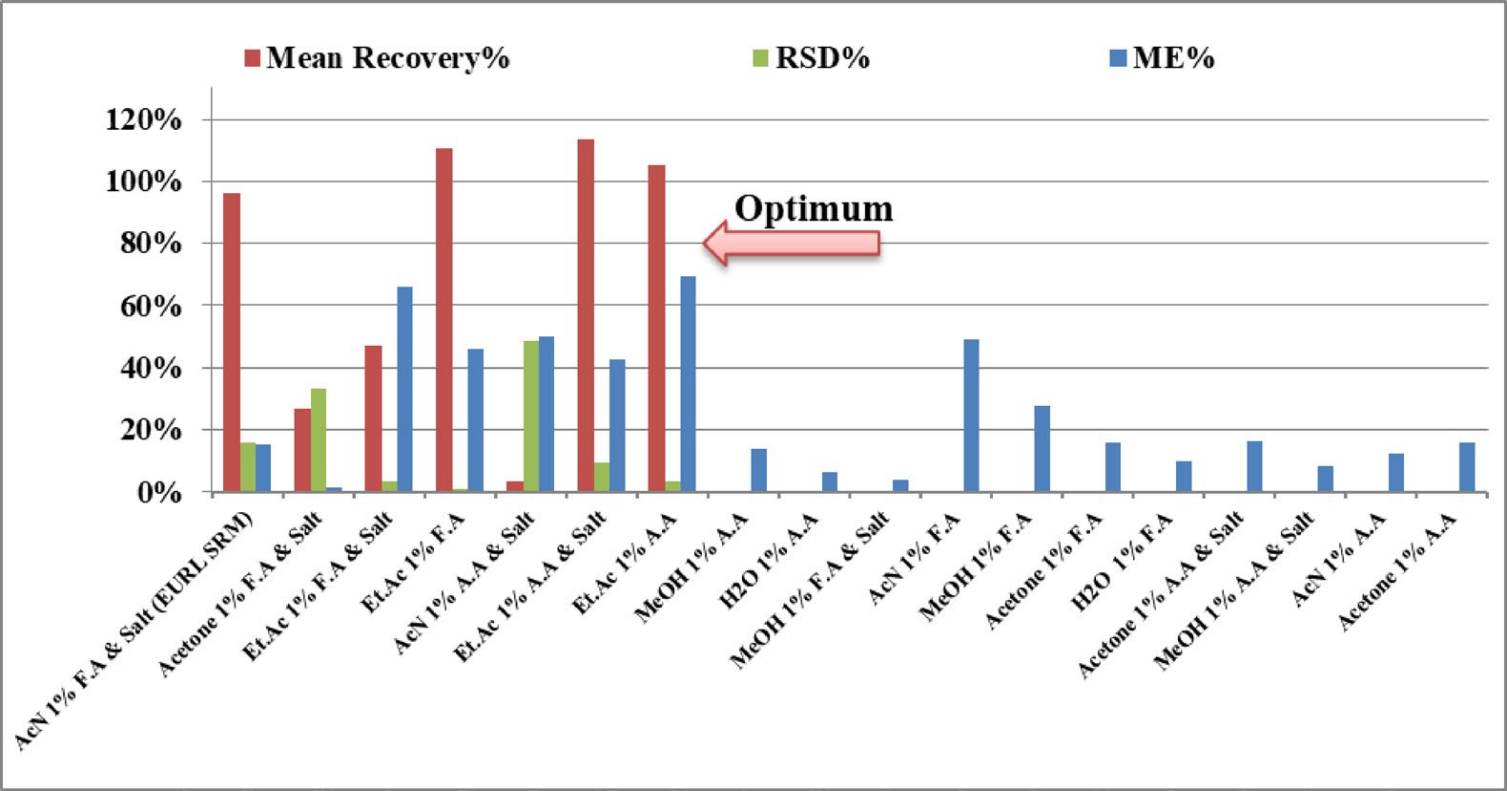
Comparison of extraction recovery, precision (RSD%), and matrix effects (ME%) using different solvent systems and additives for DI in spiked spinach samples (0.1 µg/g, 3 replicates, 18 experiments.

The extraction protocol was optimized and performed using blank spinach samples, spiked at a concentration of 0.1 µg/g. Each extraction experiment was conducted in triplicate (*n* = 3). A total of 18 different extraction experiments were tested using various combinations of solvents, acid additives, salting-out agents, different shaking times and different injection volumes. A full summary of the extraction experiments (solvent, acid additive, salt presence, recovery %, RSD%, and matrix effect %) is presented in Table 4 and visualized in Fig. 1. The detailed extraction procedure is as follows:

1. **Sample preparation**: Homogenized blank spinach samples (10 ± 0.1 g) were weighed into 50 mL polypropylene centrifuge tubes.
2. **Spiking**: Samples were fortified with dithianon standard solution to reach the target concentration of 0.1 µg/g. Samples were allowed to equilibrate for 30 min at room temperature before extraction.
3. **Solvent addition**: Ten mL of the selected extracting solvent was added. Solvents tested include: Acetonitrile (AcN), Ethyl acetate (Et.Ac), Acetone, Methanol (MeOH) and Deionized Water (DIW). Each solvent was tested with or without the addition of 1% formic acid (F.A) or 1% acetic acid (A.A).
4. **Salting-out addition**: In specific experiments, a salt mixture (4 g MgSO₄ + 1.5 g NaCl) was added immediately after solvent addition to enhance phase separation according to EURL-SRM.
5. **Shaking**: Tubes were shaken vertically using a mechanical shaker at 500 rpm for the defined shaking time in the range of 1, 5, 10, 15 and 20 min depending on the operated experiment.
6. **Centrifugation**: Samples were centrifuged at 4000 rpm for 5 min.
7. **Supernatant collection**: One mL of the upper organic layer was collected, filtered through a 0.25 μm PTFE syringe filter, and transferred to LC-MS/MS vials for analysis.
8. **LC-MS/MS analysis**: Samples were analyzed using LC-MS/MS under the conditions described in the instrumentation section.

### Validation protocol

The analytical method was validated in accordance with SANTE/11,312/2021 and Eurachem (2014) guidelines. The validation covered the following parameters: selectivity, linearity, limit of quantification (LOQ), recovery, precision, matrix effect (ME), repeatability, reproducibility, and measurement uncertainty. All validation experiments were performed under repeatable conditions and results are summarized in Tables (4, 5, 6, 7, 8, 9, 10).

1. **Blank selection and selectivity**: Six blank samples (spinach, lettuce, apple, and orange) were analyzed to confirm the absence of interfering peaks at the retention time (Rt = 4.6 min) of the target analyte. Only blank samples showing no detectable peaks were selected for spiking and further validation.
2. **LOQ determination**: LOQs were determined as the lowest spiking levels providing acceptable SANTE guideline criteria with signal-to-noise ratio ≥ 10:1.
3. **Linearity**: Calibration curves were prepared at least 6 concentration levels covering the MRLs and LOQs in both solvent and matrix-matched conditions. The acceptable SANTE criteria for the method linearity should operate at r² > 0.99.
4. **Matrix Effect (ME)**: Matrix-matched calibration curves were prepared in each blank matrix to show the ME by enhancement or suppression comparing with the solvent calibration curve. MEs were calculated by using standard addition technique as the ratio between the measured and expected concentrations. Suppression or enhancement effects were quantified from the following equation^44^:

1 $$\:\:\text{M}\text{E}{\%}=\frac{\text{X}\text{m}}{\text{X}\text{e}}\:{\times}\:100$$

where:

Xm = Measured concentration of DI added to the blank matrix.

Xe = Expected and prepared concentration of DI added to the blank matrix.

5. **Accuracy and precision**: Accuracy was evaluated as recovery at three spiking levels at (LOQ, 5 x LOQ, and 10 x LOQ µg/g) with six replicates (*n* = 6) for each level per matrix. Precision was expressed as RSD%. The calculated average recoveries with RSDs were accepted according to the SANTE guideline criteria if the recovery ranged from 70 in 120% and RSDs less than or equal 20%^44^.

2 $$\:\text{S}\text{p}\text{i}\text{k}\text{e}\:\text{R}\text{e}\text{c}\text{o}\text{v}\text{e}\text{r}\text{y}{\%}\:\:=\frac{\text{S}\text{m}}{\text{S}\text{e}}\:{\times}\:100$$

where:

Sm = Measured concentration of DI in the spiked samples by a blank matrix.

Se = Expected and prepared concentration of DI in the spiked samples by a blank matrix.

6. **Repeatability and reproducibility**: Repeatability and reproducibility were assessed by calculating pooled RSD% across validation levels. Reproducibility was determined by analyzing 12 replicates (*n* = 12) over 12 days by four analysts using the same instrument. RSDwR values were acceptable when RSDwR ≤ 20%. The pooled relative standard deviations were calculated from the following equation according SANTE guideline.
  3 $$\text{RSD}\:\text{pool}=\text{SORT}([\text{(n1-1)}\times\text{RSD12}+\text{(n2-1)}\times\text{RSD22}+\dots]/[\text{(n1-1)}+\text{(n2-1)}+\dots])$$

where:

RSD = Relative standard deviations of DI for spiked samples at all different validation concentration levels.

n = Number of replicate tests of DI for spiked samples for each concentration level.

7. **Measurement Uncertainty**: Expanded uncertainty (Uexp) was calculated based on both bias and precision contributions according to SANTE 2021 equations. Final Uexp within the acceptable measurement uncertainty at 50% limit. The measurement uncertainty was determined using the following formulas:

4 $$\:\text{R}\text{e}\text{l}\text{a}\text{t}\text{i}\text{v}\text{e}\:\text{B}\text{i}\text{a}\text{s}{\%}\:=\frac{\text{C}\text{m}-\text{C}\text{s}\text{p}}{\text{C}\text{s}\text{p}}\:\times\:100$$

5 $$\:\text{R}\text{S}\text{D}\text{w}\text{R}{\%}\:=\frac{\text{S}\text{D}.\text{S}}{\text{M}\text{e}\text{a}\text{n}\:\text{C}\text{o}\text{n}\text{c}\text{e}\text{n}\text{t}\text{r}\text{a}\text{t}\text{i}\text{o}\text{n}}\:\times\:100$$

6 $$\:\text{U}\:\left(\text{b}\text{a}\text{i}\text{s}\right)\:=\sqrt{\left(Mean\:bias\right)2+(SD.P\:bias)2}$$

7 $$\:\text{U}\text{(Precision)}=\text{RSDwR}{\%}$$

8 $$\:\text{U}\text{c}\text{o}\text{m}\:\:=\sqrt{\left(Mean\:bias\right)2+\left(SD.P\:bias\right)2+\left(\text{R}\text{S}\text{D}\text{w}\text{R}\right)2}$$

9 $$\:\text{Uexp}=\text{k}\:\text{x}\:\text{Ucom}-2\times\text{Ucom}$$

Where :

Cm: Measured Concentration.

Csp: Spiked Concentration.

RSDwR: Within-Laboratory Reproducibility relative standard deviation.

U (bais): Uncertainty component for the bias.

U (Precision): Uncertainty component for the precision.

Ucom: Combined Uncertainty.

Uexp: Expanded Uncertainty.

k: Coverage factor k = 2 for 95% confidence.

n: Number of replicates.

### Final optimized single-phase extraction method

Ten grams of fresh samples were weighed into a 50 ml polypropylene falcon centrifuge tube. After that, 10 ml of acidified ethyl acetate containing 1% Acetic acid was dispensed into each sample. Shaking the samples was carried out for 15 min at 500 rounds per minute (rpm) in an automatic axial agitator (Geno/Grinder). Then, the tubes were centrifuged for 5 min at 4500 (rpm). Finally, about 1.0 ml of the upper layer was filtered through a PTFE syringe filter and transferred into glass vials to be ready for the direct analysis using LC-MS/MS.

## LC ESI (-) MS/MS instrumental analysis

The separation of the target analytes was carried out using an Exion LC Shimadzu HPLC system, equipped with a C18 column (proshell 120, 3 mm×50 mm×2.7 μm). The separation was achieved by using a mobile phase comprising two components: (A) a mixture of DIW: MeOH (9:1, v: v) with a pH of 4, containing ammonium formate as a solvent; and (B) pure MeOH. The mobile phase was pumped at a flow rate of 0.4 ml/min, and an optimized solvent gradient program was used as shown in Table 1. The retention time for dithianon was found to be 4.6 min. This chromatographic method was able to separate dithianon peaks effectively in complex matrices, without requiring a clean-up step.

**Table 1:** HPLC binary gradient parameters including time per min, flow rate, mobile phase (A) a mixture of DIW: MeOH (9: 1, v: v) with a pH of 4, containing ammonium formate buffer as mobile phase buffer; and mobile phase (B) a pure methanol as mobile organic phase.

Time (min.)	Flow (mL/min.)	Mobile phase (A) %	Mobile phase (B) %
0	0.4	85	15
1	0.4	85	15
3	0.4	10	90
5	0.4	10	90
7	0.4	0	100
9	0.4	85	15
12	0.4	85	15

AB SCIEX 6500 plus equipped with an Ion Drive Turbo V source was used in this study. It was operated in the soft negative electrospray ionization (ESI -ve) mode. Apparatus syringe pump has been used to introduce individual dithianon solution with a concentration of 0.1 µg/ml in mobile phase buffer: methanol (1:1 v/v) into only the activated MS instrument to make tuning for the MS/MS parameters. An automated mass infusion was applied to optimize mass parameters of the most sensitive multiple reaction monitoring (MRM) transitions for dithianon. The primary mass parameters that were optimized for this study included the entrance potential (EP), declustering potential (DP), collision energy (CE), and collision cell-exit potential (CXP) as shown in Table 2. Additionally, other mass parameters, such as the ion-spray voltage (IS) − 4500 v, ion source temperature (TEM) 450 °C, curtain gas (CUR) 20.0, collision gas (CAD) medium, nebulizing gas (GS1) 45, and drying gas (GS2) 50 were automatically optimized by LC-MS/MS. In the quadrupoles of our LC-MS/MS, the most sensitive fragments were chosen to improve the selectivity and sensitivity of detected ions. These parameters have been collected to build up the acquisition method for determination of dithianon residues.

**Table 2:** LC-MS/MS optimization parameters including molecular masses to charge ratio (m/z) in the quads (q1&q3), molecular ion, declustering potential (DP), entrance potential (EP), collision energy (CE) and collision cell exit potential (CXP) for the four targeted MRM transitions.

Pesticide name	Type	Q1 Mass (Da)	Q3 Mass (Da)	Rt (min)	DP (Volt)	EP (Volt)	DP (Volt)	CE (Volt)	CXP (Volt)
Dithianon-1	Quantifier	296	264	4.6	−130	−10	−30	−5	−5
Dithianon-2	Qualifier	296	240	4.6	−130	−10	−28	−5	−5
Dithianon-3	Qualifier	296	238	4.6	−130	−10	−30	−1	−1
Dithianon-4	Qualifier	296	164	4.6	−130	−10	−28	−5	−5

## Results and discussion

Dithianon pesticide was problematic compound in the universal QuEChERS extraction method so, we tried to extract and purify its residues in fruits and vegetables with various modifications. The efficiency of the extraction method was developed by applying different extracting solvents, and shaking time and to get the optimum conditions for the extraction method.

### Method optimization

Development of our LC-MS/MS injection method was carried out by optimizing the mass physical parameters during the automatic optimization for the dithianon compound on LC-MS/MS device in addition to optimization of mobile phase compositions, ionization mode, and injection volume to get the best mass parameters and good chromatographic separation.

### Optimization of ionization in LC-MS/MS

As shown in Table 3, Dithianon is a fungicide and not typically characterized by a pKa value in the way that acidic or basic compounds are following. Its chemical structure, which includes sulfur and chlorine atoms, doesn’t feature functional groups that would usually confer strong acidic or basic properties so, dithianon is a neutral compound and does not exhibit the typical properties of acids or bases according to its molecular structure that can be ionized by using negative or positive electrospray ionization modes ESI (- or +) because it can release proton and form negative (deprotonated) adducts [M-H]^−^ or can accept proton to form positive (protonated) adducts [M + H]^+^ but in our results when we compare between the last 2 cases we found that deprotonated adducts have been more sensitive than protonated adducts where the 2 calibration levels with concentrations 0.001 and 0.002 µg/ml were not detected in ESI (+) in addition to the calibration level with concentration 0.5 µg/ml in ESI (-) has peak area and peak height larger than them in ESI (+). Therefore, the use of an acidic mobile phase containing formate anion in negative ion mode improved ESI (-) responses for DI acidic compound.

**Table 3:** The list of some important properties for DI.

Pesticide name	Dithianon
Molecular Structure	
Molecular Weight	296.3 g/mol
Molecular Formula	C_14_H_4_N_2_O_2_S_2_
IUPAC Name	5,10-dihydro-5,10-dioxonaphtho[2,3-b]−1,4-dithiine-2,3-dicarbonitrile
CAS No.	3347-22-6
Chemical Class	Quinone compound
Mode of Action	Fungicide
Stability	Decomposed by alkaline media, concentrated acids, and prolonged heating
Acid Dissociation Constants (P^Ka^)	No dissociation in water
Octanol/Water Partition Coefficient (log p^Kow^)	3.2 (20 °C at pH 2)

### Optimization of extracting solvent

Dithianon has neutral and mid-polar properties so, we need to add suitable acid such as formic or acetic acid into the extracting solvent to increase the abundance of its non-ionized form which facilitate the mission of the extracting organic solvent to extract our organic compound.

Optimization of the extraction method was done by using different extracting solvents such as (Acetonitrile, Methanol, Ethyl acetate, water, or Acetone), different additives such as (1% Formic acid or 1% Acetic acid), and salting out powder (with or without MgSO4 and NaCl powder) in 18 experiments.

Blank spinach samples were spiked by different extracting solvents at 3 replicates at concentration 0.1 µg/g which were analyzed using LC-MS/MS to evaluate the extraction efficiency. (The term salt refers to a mixture of 4 g magnesium sulfate and 1.5 g sodium chloride under the image). The effectiveness of various extraction solvents with different additives was evaluated by comparing their mean recovery percentages, precision (RSD%), matrix effects (ME%), and standard deviation (SD). Figure 1 presents the recovery efficiency of each solvent system tested.

Among the solvent systems tested, ethyl acetate (Et.Ac) with 1% acetic acid (A.A) exhibited the highest recovery rates, making it the most effective solvent in this study. Specifically, Et.Ac 1% A.A & Salt (Experiment 6) achieved a recovery of 113% with a relative standard deviation (RSD) of 10% and a moderate matrix effect (ME) of 43%. Similarly, Et.Ac 1% A.A (Experiment 7) yielded a recovery of 105%, a very low RSD of 3%, and a matrix effect of 70%. These results suggest that ethyl acetate with acetic acid provides a suitable balance between extraction efficiency and reproducibility. The slightly higher matrix effect when salt was omitted suggests that the presence of salt may help mitigate some matrix interference in certain conditions, but this comes with a trade-off in recovery precision.

Acetonitrile (AcN) with 1% formic acid (F.A) and salt (Experiment 1), following the EURL SRM protocol, also performed well, with a mean recovery of 96%, an RSD of 16%, and a matrix effect of 15%. While the recovery was slightly lower than that of the Et.Ac based solvents, it is still a robust option with low matrix interference.

The presence of additives such as formic acid (F.A), acetic acid (A.A), and salts was critical in influencing the extraction performance. In most cases, the addition of salts resulted in either improved recovery (as seen in Experiment 1) or slightly lower recovery with better matrix effects (as seen in Experiment 6). For example, removing the salt from the Et.Ac 1% F.A system (Experiment 4 vs. Experiment 3) improved the recovery significantly, from 47% to 111%, indicating that the salt may hinder extraction in certain solvent systems. However, the trade-off came in the form of increased matrix effects (66% vs. 46%) when the salt was excluded.

The poor performance of Acetone 1% F.A & Salt (Experiment 2), which yielded only 27% recovery with high variability (RSD = 33%), further highlights the variability in the role of additives. Meanwhile, the combination of AcN 1% A.A & Salt (Experiment 5) provided almost no recovery (3%), suggesting that this combination is not suitable for the extraction of the analyte under study.

Several solvent systems failed to produce detectable peaks, indicating that they were ineffective for the extraction of the analyte. Notably, methanol (MeOH) and water (H₂O), with or without additives, yielded no peaks in multiple experiments (Experiments 8 to 18). This suggests that these solvents, either alone or with acid/salt additives, are not capable of efficiently extracting the analyte from the matrix.

While high recovery is desirable, it is essential to ensure precision and minimal matrix interference. Both Et.Ac 1% A.A systems (Experiments 6 and 7) demonstrated low RSD values (10% and 3%, respectively), indicating good reproducibility. The matrix effects in these cases were moderate to high, but overall acceptable given the high recoveries achieved. AcN 1% F.A & Salt (Experiment 1) had one of the lowest matrix effects (15%) but a relatively higher RSD (16%), suggesting slightly lower precision despite low matrix interference.

Based on the Fig. 1 results, Et.Ac 1% A.A with or without salt proved to be the most effective solvent system for the extraction of the target analyte, offering both high recovery and acceptable precision. Acetonitrile 1% F.A & Salt, as per the EURL-SRM, also delivered strong results, albeit with slightly lower recoveries. The study demonstrates the importance of optimizing both solvent type and additive combinations to achieve optimal extraction efficiency.

### Optimization of shaking time

**Fig. 2:**
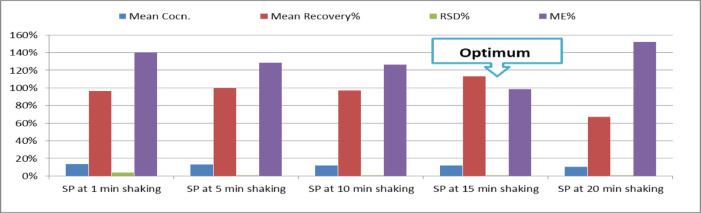
Optimization of shaking time using of spiked spinach matrix by 0.1 µg/g DI as expected value at 5 different shaking times in triplicates (*n* = 3).

The optimization of shaking time is an important step in the homogenization of samples for analytical purposes. The appropriate shaking time will ensure that the sample is thoroughly mixed and homogenized, leading to more accurate and precise results. To optimize shaking time, a series of experiments were performed using different shaking times 1, 5, 10, 15–20 min for triplicate (*n* = 3) spike quality control (SP-QC) blank spinach samples at concentrations 0.1 µg/ml. The impact of varying shaking times on mean concentration, recovery percentage, relative standard deviation (RSD%), and matrix effects (ME%) was evaluated, and the findings are detailed below:

According to Fig. 2, The mean concentration of the analyte decreased with increased shaking time, from 0.136 at 1 min to 0.102 at 20 min. This decrease suggests that longer shaking times may lead to the analyte reaching closer to the true value of the concentration at 0.1 µg/g.

Recovery percentages varied significantly with shaking time. Recovery was 97% at 1 min, increased to 113% at 15 min, and then dropped to 67% at 20 min. The increase in recovery percentage at 15 min suggests a period of optimal interaction between the analyte and the matrix. However, the substantial decrease at 20 min indicates potential issues such as analyte degradation or exacerbated matrix interference over extended durations.

The RSD% values were low across all shaking times, ranging from 0.1% to 4%, reflecting high precision in the measurements. This consistent precision indicates that the analytical method is reliable and reproducible regardless of shaking time.

Matrix effects were highest at 1 min (141%) and increased further to 152% at 20 min. This trend indicates significant matrix interference at both the shortest and longest shaking times. The matrix effects decreased to 98% at 15 min, suggesting that this duration minimizes matrix interference more effectively compared to other times.

Based on the data in Fig. 2, Fifteen minutes is identified as the optimal shaking time. This duration provides a favorable balance of the following:

1. Recovery Percentage: Highest at 113% at 15 min, indicating improved accuracy.
2. Matrix Effects: Lowest at 98%, suggesting reduced matrix interference compared to other shaking times.
3. Mean Concentration: Although it is lower than at shorter times, the concentration at 15 min is more stable compared to the significant drop observed at 20 min.
4. RSD%: Remains low and consistent across all shaking times, underscoring the precision of the method.

In conclusion, the 15-minute shaking time offers an optimal compromise by balancing high recovery percentage, minimized matrix effects, and stable mean concentration. This shaking time is recommended to ensure accurate and reliable analytical results while mitigating matrix interference.

### Optimization of injection volume

Optimization of the injection volume is important in order to achieve a good chromatogram with optimal characteristics such as high intensity, peak shape, recovery, and low relative standard deviation. These factors contribute to obtaining more accurate and precise results. In order to optimize the injection volume, a series of experiments were conducted using different injection volumes of 10, 5, 3, or 2 µL for triplet spiked quality control (SP-QC) blank spinach samples with an expected concentration of 0.1 µg/ml.

**Fig. 3:**
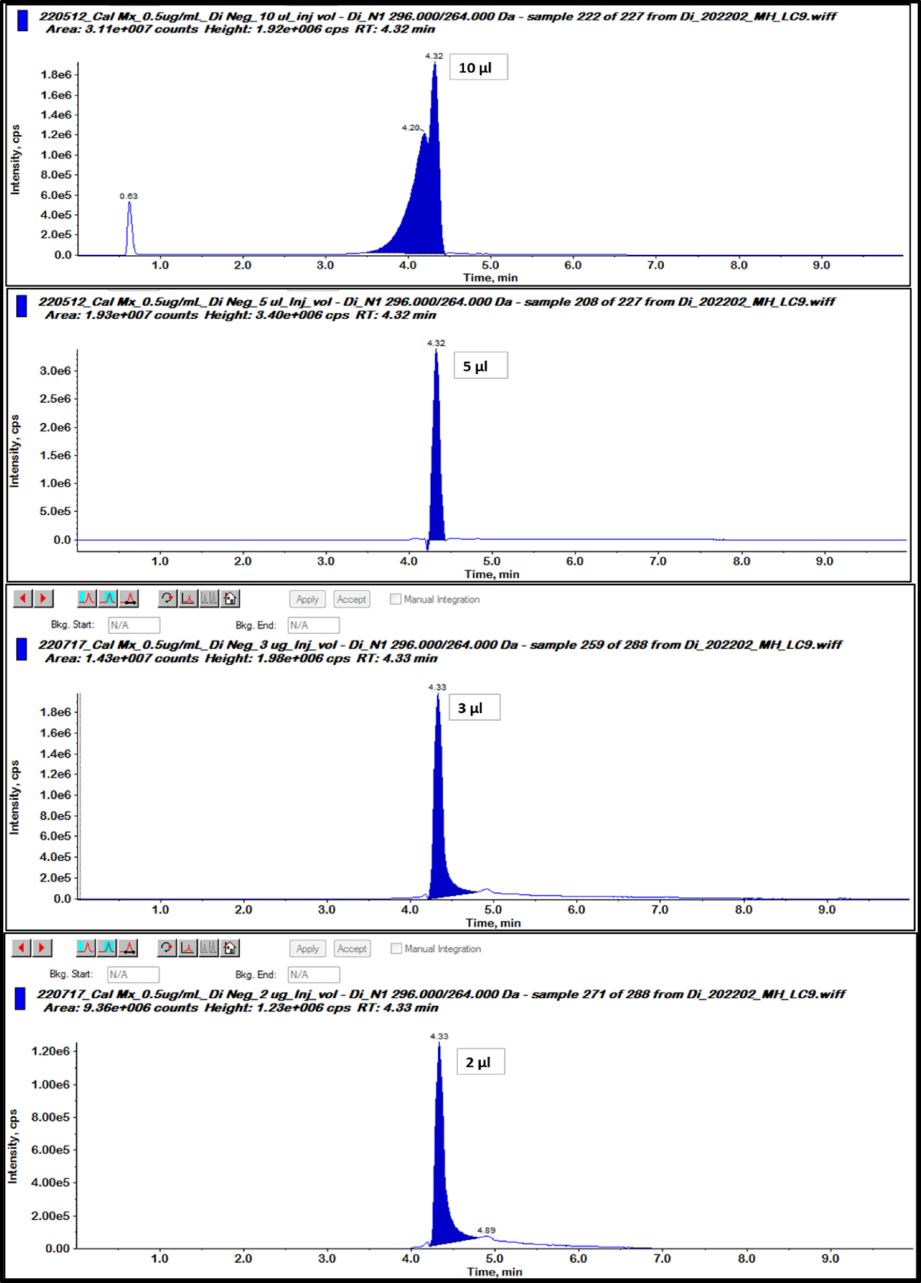
Optimization of injection volume using the LC-MS/MS chromatograms of spiked spinach matrix by 0.1 µg/g DI as expected value at 4 different injection volumes (10, 5, 3 and 2 µl) in 3 replicates.

As described in Fig. 3 bad peak shape was found due to chromatographic peak splitting and broadening at injection volume equal to 10 µL so, ten microliter injection volume was ignored. Although 5 µL injection volume gave accepted recovery %, RSD%, and good peak shape, large injection volume at 5 µL may be make contamination of our quadruple of MS instrument and make carry over in the next injected samples so, five microliter injection volume was excluded. Following the SANTE/11,312/2021 guideline, the accepted average recovery percentage was found to be 81%, and the relative standard deviation (RSD%) was at 10% by using injection volume of 3 µL on the other side, injection volume 2 µL gave bad average recovery percentage at 59% with RSD% equal to 13%. Thus, optimal injection volume was found to be at 3 µl.

### Method validation

The purpose of analytical method validation is to ensure the quality, accuracy, and comparability of analytical method results. This helps to increase confidence in the analytical method and ensure compliance with ISO/IEC 17,025 standards. Our in-house validation process includes testing various parameters such as sensitivity, limit of quantitation (LOQ), linearity, matrix effect (ME), recovery, precision, repeatability and reproducibility. The guidelines used for the validation process were primarily from the most recent versions of Eurachem/2014^45^ and SANTE/11,312/2021guidelines^46^ following the procedures outlined in EU regulations.

### Selectivity

**Fig. 4:**
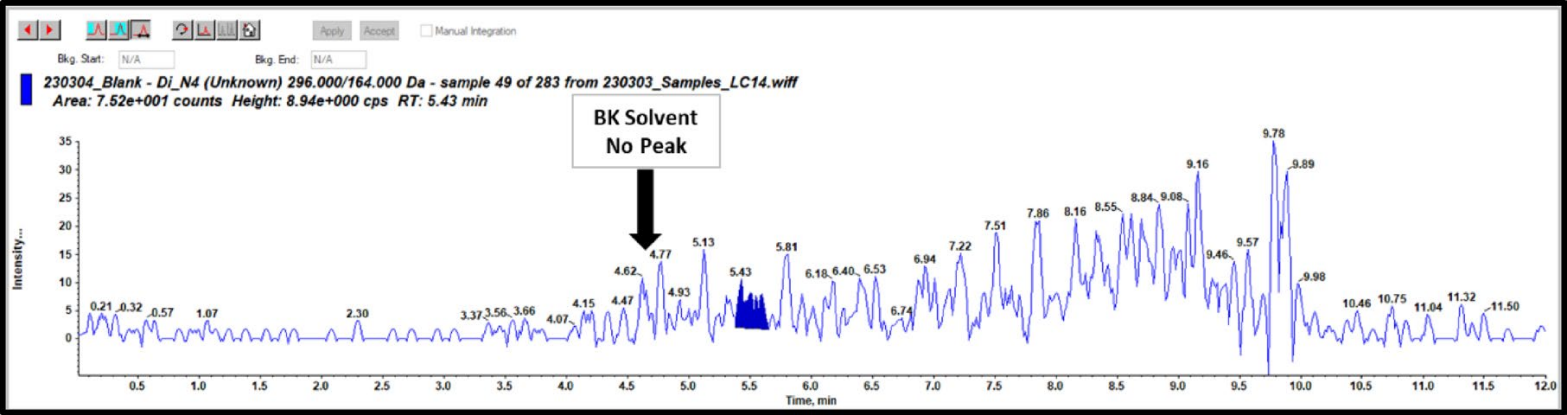
LC-MS/MS Chromatogram of blank Reagent.

**Fig. 5:**
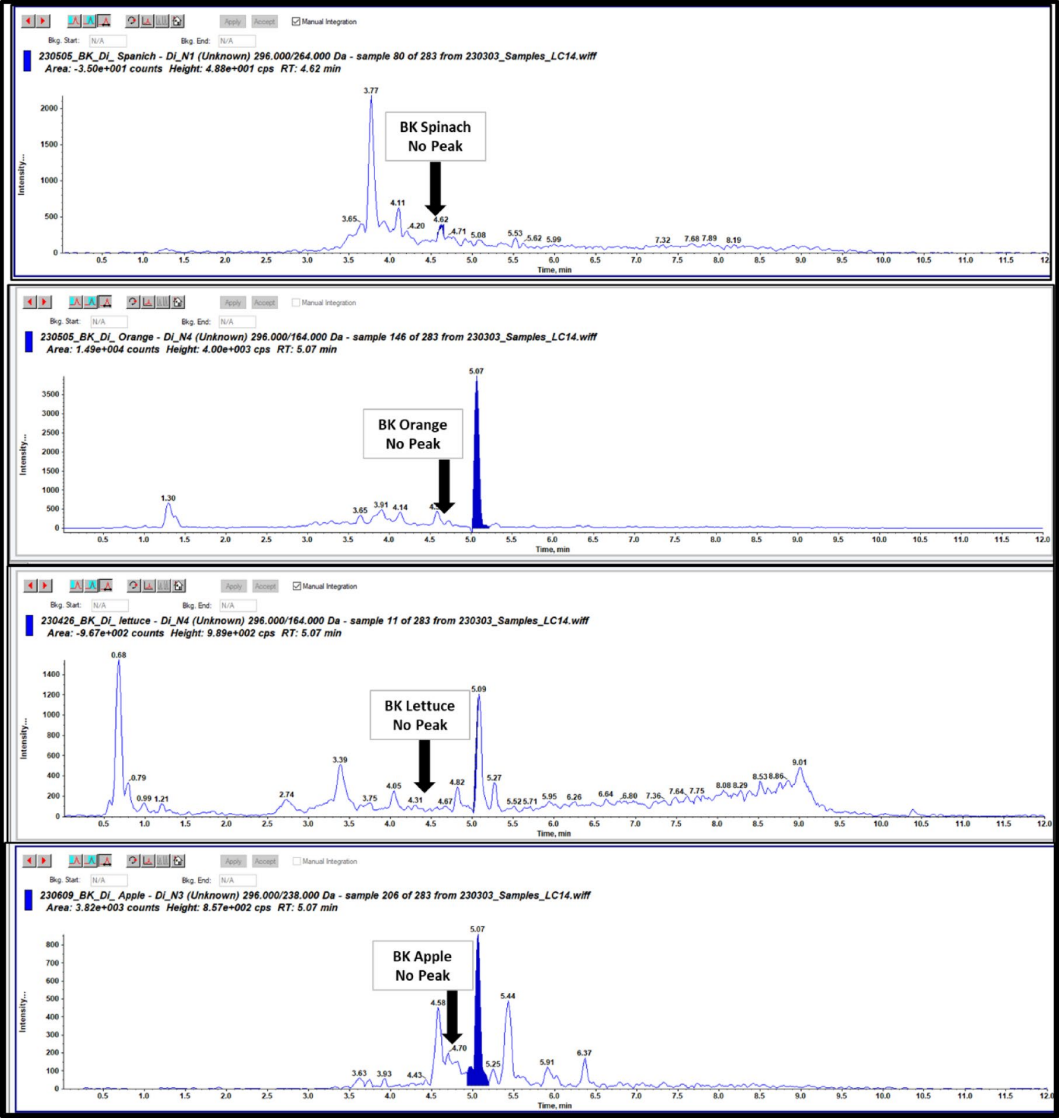
LC-MS/MS Chromatograms of blank apple, spinach, lettuce and orange matrices.

Selectivity test was operated to test if there are any possible interference for our targeted DI pesticide from the used solvents, chemicals, reagents, tools, apparatus, mobile phase or tested matrix. As shown in Figs. 4 and 5 There were no interfering peaks for DI at its retention time which equal to 4.6 min in the chromatograms for one blank methanol solvent and four blank matrices such as lettuce, spinach, apple, and orange.

### Limit of quantification (LOQ)

Table 4 summarizes the details of the developed method, including the Limit of Quantitation (LOQs), which represents the minimum concentration that can be accurately detected and quantified using the analytical technique that were ranged from 0.01 to 0.05 µg/g. Additionally, this table provides information on the lowest calibration level (LCL) concentrations which represent the minimum concentrations used in the calibration process of the analytical method that equal to 0.001 µg/ml.

**Table 4:** List of lowest calibration level (LCL) concentrations, correlation variation (r2), retention time (Rt) by minutes and European maximum residue limits (EU MRLs) by (µg/g) for apple, spinach lettuce, and orange.

Parameters	DI criteria and optimization factors				Optimum Criteria
LCL in solvent (µg/ml)	0.001				
r2 in solvent	0.988				
Rt (min.)	4.6				
LOQ in EURL-SRM (µg/g)	0.1				
LOQ (µg/g)	0.05 (apple)	0.05 (spinach)	0.01 (lettuce)	0.05 (orange)	
EU MRLs (µg/g)	3 (apple)	0.6 (spinach)	0.01 (lettuce)	1 (orange)	
Extracting Solvents (10 ml)	methanol, acetonitrile, ethyl acetate and water				ethyl acetate
Additives adding	Without additives, 1% acetic acid and 1% formic acid				1% acetic acid
Salting out (4 g MgSO4 & 1.5 g NaCl)	With salting out and without salting out				without salting out
Injection Volumes (µL)	10, 5, 3 and 2				3
Shaking time (min)	1, 5, 10, 15 and 20				15

LCL used as reference points for determination of the sensitivity of our LC-MS/MS instrument for the tested DI pesticide residues. Our detection method can quantify DI concentrations less than or equal to the values for European Union Maximum Residue Limits (EU MRLs) for DI in some fruits and vegetables food.

**Fig. 6:**
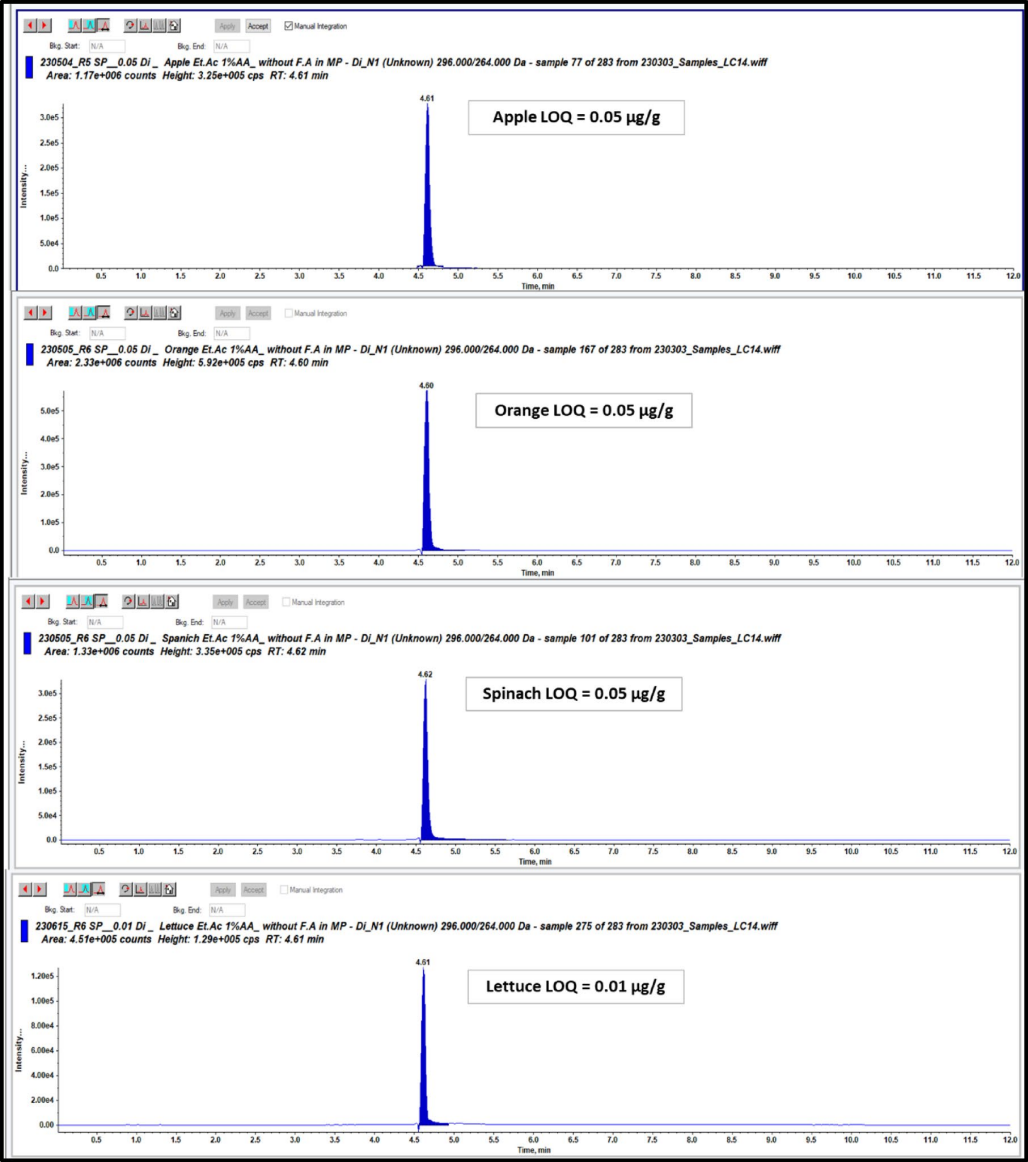
LC-MS/MS Chromatograms for DI at LOQs for apple, spinach, lettuce and orange matrices.

Our study focused on determining the LOQs for DI in various food matrices. The LOQs were expressed as the lowest validation level according to SANTE guideline where six spiked blank commodities such as spinach, lettuce, apple, and orange at concentration 0.01 µg/g for lettuce and 0.05 µg/g for spinach, apple, and orange were injected on LC-MS/MS in which the out-put chromatograms have a signal-to-noise ratio of more than or equal to 10:1. Figure 6 displays the LC-MS/MS chromatograms depicting DI at LOQ levels in spinach, lettuce, apple, and orange matrices. Our developed method demonstrated a significantly enhanced sensitivity for DI and compared to the reference approach recommended by the EURL. As presented in Table 4, the LOQs were well below the relevant MRLs (which range from 0.01 to 3 µg/g for the matrices studied). Specifically, for lettuce commodity with an MRL of 0.01 µg/g, the method’s LOQ is equal to the MRL, which is acceptable and fit-for-purpose for compliance testing according to SANTE guidelines, which require an LOQs ≤ the MRLs. Specifically, LOQs achieved by our method were more than 2–10 times the LOQs established in the EURL method, as described in Table 10. This substantial improvement highlights the robustness and efficiency of our single-phase extraction procedure and its suitability for trace-level monitoring of DI residues in food matrices. By providing a markedly lower LOQ, our single-phase extraction method not only ensures higher reliability in residue detection but also offers a more powerful tool to present a significant advantage over more complex methods that achieve similar sensitivity for regulatory compliance and consumer safety assurance.

### Linearity

**Fig. 7:**
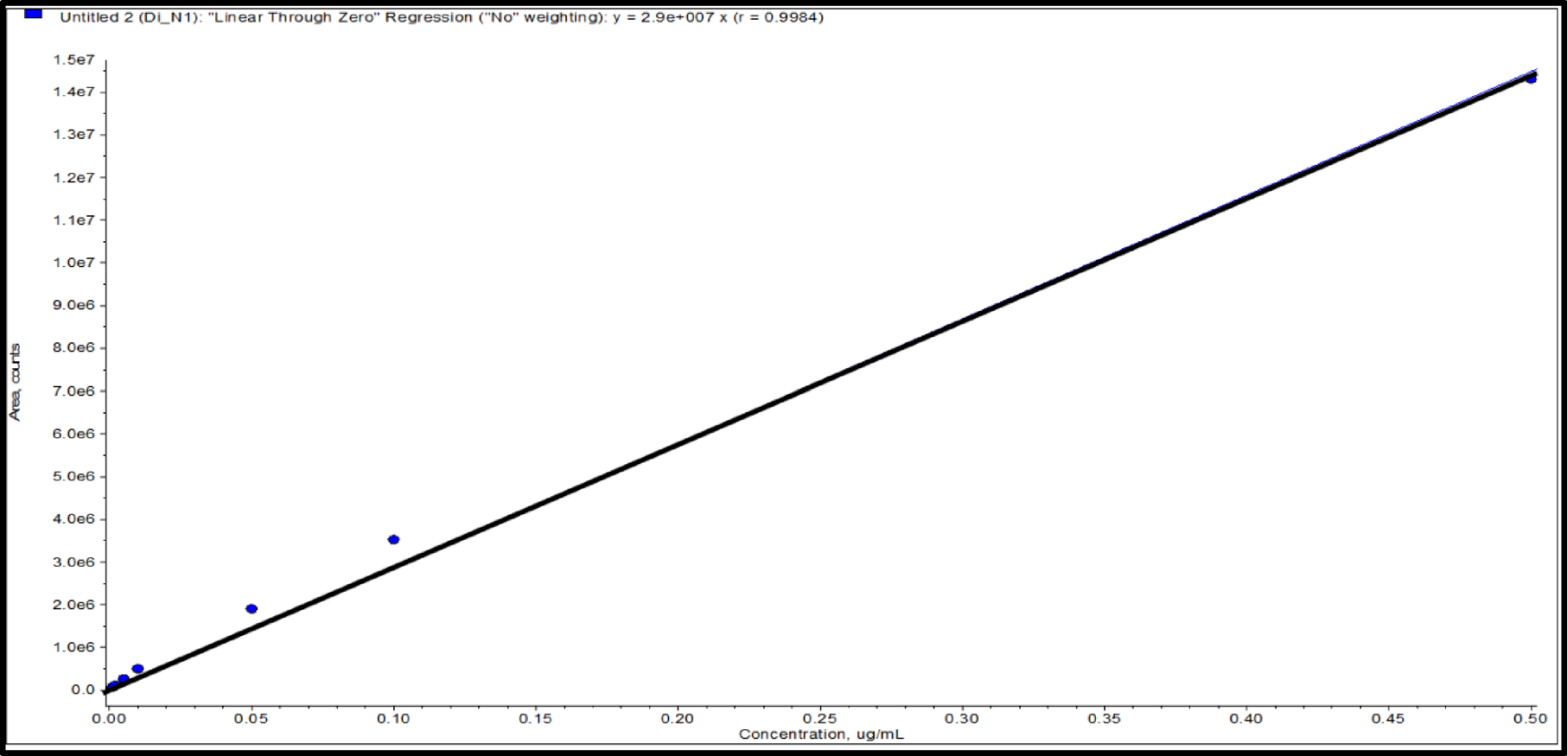
LC-MS/MS calibration curve for DI in solvent.

Calibration curve is the relationship between different calibration levels with concentrations of 0.001, 0.002, 0.01, 0.05, 0.1, and 0.5 µg/ml for DI and the corresponding LC-MS/MS peak areas of DI by counts. The correlation variation (r^2^) indicates the strength of the correlation between the measured values and the expected values. A high correlation coefficient closed to 1 suggests a strong relationship between the measured and expected values. Figure 7 illustrates the linear calibration curve for DI with accepted regression coefficient (r^2^) at 0.998, exceeding the acceptable threshold of 0.99 for a linear calibration curve according to the SANTE guideline. The LC-MS/MS quantitation method was utilized, employing peak area regression parameters with a none weighting curve and passing through the original zero points with linear shape.

### Matrix effect (ME)

The phenomenon matrix effect in analytical chemistry refers to the influence of matrix components present in a sample on the accuracy and reliability of the analysis. During the analysis of our targeted analyte DI, the matrix components can affect on DI concentration which can lead to either suppression or enhancement of the signals obtained during analysis^47^.

Our study in ME was carried out by using matrix-matched calibration technique where DI with different concentration levels at 0.05, 0.1, and 0.5 µg/ml were prepared in blank extracts of spinach, apple, lettuce, or orange matrix.

**Fig. 8:**
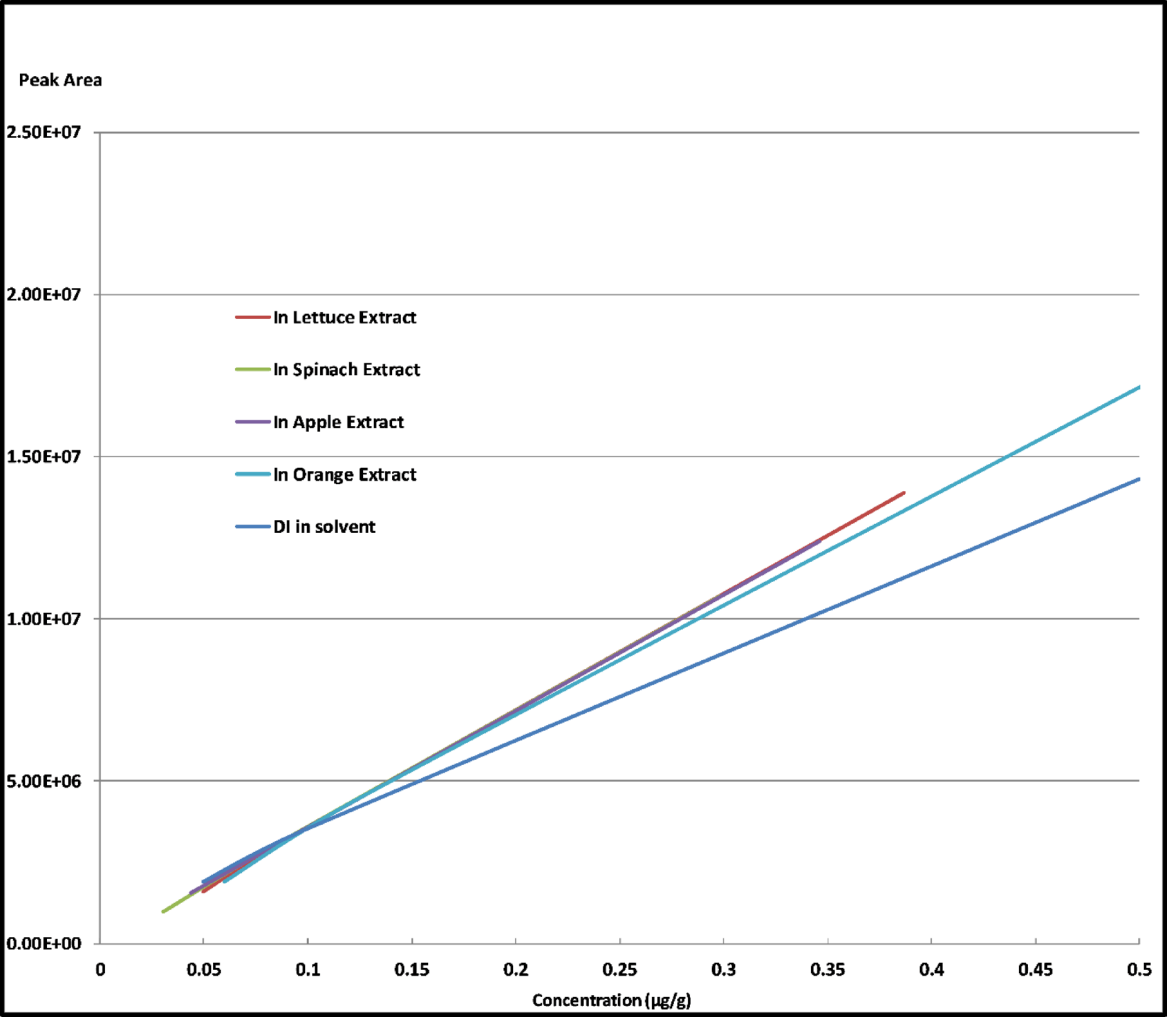
LC-MS/MS calibration curves for DI in solvent and apple, lettuce, spinach and orange extract matrices.

As shown in Fig. 8 Calibration curves were generated using both a solvent and the extract matrix from the different matrices. By comparing the linear calibration curves in Methanol and in blank extract of each tested commodity, we could be able to assess the impact of the matrix on the signals of DI at lower concentration levels.

It was observed that the presence of spinach and apple matrices caused ion suppression, meaning that the signals and the found concentrations of DI were reduced compared to the signals obtained from them in Methanol solvent. On the other hand, the presence of orange and lettuce matrices resulted an enhancement effect, where the signals and the found concentrations of DI were enhanced.

The matrix effect percentage (ME%) can be calculated according to the last equation (1) (SANTE/11312/2021) by making single point standard addition technique at definite concentration.

### Accuracy and precision

The accuracy and precisions were determined by calculating the recovery (Rec%) and relative standard deviation (RSD%) according to Eurachem and SANTE Guidelines (Eurachem Guideline 2014; SANTE/11312/2021). The recovery percentage is calculated from the last equation (2).

In the mentioned study, recovery and precision samples were conducted by spiking blank samples of four different food commodities at concentration levels of 0.05, 0.1, and 0.5 µg/g. This means that for each commodity, there were 18 spiked samples at the 3 different concentration levels.

The output data from the analytical method validation were presented in Tables 5, 6, 7 and 8. These tables show that the average recoveries for the target analyte were within an acceptable range of 85–113% with RSDs % ≤ 8% for all tested food commodities, including spinach, lettuce, apple, and orange. The acceptable recovery range indicates that the analytical method used is capable of accurately measuring the concentration of dithianon in these representative food commodities.

**Table 5:** The average spiked recovery%, standard deviation (SD) and relative standard deviation (RSD%) results of DI by LC-MS/MS that were spiked at 0.05, 0.1, and 0.5 µg/g (6 replicates, one analyst, at each level) in Apple matrix.

Spiked Recovery% Replicates	SP Level 0.05 µg/g	SP Level 0.1 µg/g	SP Level 0.5 µg/g
R1	93%	103%	105%
R2	86%	97%	100%
R3	83%	107%	97%
R4	83%	98%	100%
R5	82%	95%	95%
R6	93%	85%	97%
Average Spiked Recovery%	87%	98%	99%
SD (µg/g)	0.051	0.075	0.035
RSD%	6%	8%	4%

**Table 6:** The average spiked recovery%, standard deviation (SD) and relative standard deviation (RSD%) results of DI by LC-MS/MS that were spiked at 0.05, 0.1, and 0.5 µg/g (6 replicates, one analyst, at each level) in spinach matrix.

Spiked Recovery% Replicates	SP Level 0.05 µg/g	SP Level 0.1 µg/g	SP Level 0.5 µg/g
R1	113%	94%	108%
R2	118%	84%	97%
R3	112%	83%	98%
R4	113%	89%	85%
R5	104%	77%	101%
R6	115%	80%	96%
Average Spiked Recovery%	113%	85%	98%
SD (µg/g)	0.047	0.062	0.075
RSD%	4%	7%	8%

**Table 7:** The average spiked recovery%, standard deviation (SD) and relative standard deviation (RSD%) results of DI by LC-MS/MS that were spiked at 0.05, 0.1,0.05 and 0.01 µg/g (6 replicates, one analyst, at each level) in lettuce matrix.

Spiked Recovery% Replicates	SP Level 0.05 µg/g	SP Level 0.1 µg/g	SP Level 0.5 µg/g	SP Level 0.01 µg/g
R1	111%	114%	104%	93%
R2	116%	110%	109%	98%
R3	116%	111%	112%	96%
R4	108%	102%	115%	97%
R5	116%	103%	102%	93%
R6	113%	103%	110%	93%
Average Spiked Recovery%	113%	107%	109%	95%
SD (µg/g)	0.033	0.051	0.049	0.023
RSD%	3%	5%	4%	2%

**Table 8:** The average spiked recovery%, standard deviation (SD) and relative standard deviation (RSD%) results of DI by LC-MS/MS that were spiked at 0.05, 0.1, and 0.5 µg/g (6 replicates, one analyst, at each level) in orange matrix.

Spiked Recovery% Replicates	SP Level 0.05 µg/g	SP Level 0.1 µg/g	SP Level 0.5 µg/g
R1	123%	107%	114%
R2	117%	105%	105%
R3	109%	111%	101%
R4	112%	111%	118%
R5	105%	105%	113%
R6	113%	109%	109%
Average Spiked Recovery%	113%	108%	110%
SD (µg/g)	0.063	0.028	0.063
RSD%	6%	3%	6%

### Repeatability and reproducibility

The repeatability is expressed as pooled relative standard deviation (RSD%) which refer to precision of the analyte measurements that were obtained by using the same method, tested commodity, materials, and equipment in a single laboratory over a short time. The pooled relative standard deviations were calculated from the last equation (3).

Repeatability test was checked by making fortification on blank samples at concentrations 0.05, 0.1, and 0.5 µg/g to obtain spiked samples for all tested commodities in addition to the validation level at concentration 0.01 µg/g for lettuce which is lower than the corresponding Maximum Residue Limits (MRLs) for dithianon in the CODEX and EURL pesticide databases as shown in Tables 5, 6, 7, 8 and 9.

**Table 9:** Repeatability LC-MS/MS data for DI that was spiked at 0.05, 0.1, and 0.5 µg/g (*n* = 6 replicates, one analyst, at each level) in apple, spinach and orange matrices in addition to the spiking level at 0.01 µg/g in lettuce Matrix. Reproducibilty LC-MS/MS data for DI that was spiked at 0.1 µg/g (*n* = 12 replicates, 4 analysts, 12 different days and in 4 matrices).

Repeatability Data		Apple	Spinach	Orange	Lettuce
	Pooled SD (µg/g)	0.053	0.06	0.056	0.041
	Pooled RSD	0.056	0.07	0.052	0.038
	Pooled RSD%	6%	7%	5%	4%
Reproducibility Data	Days	Commodities	Found Concentration (µg/g)	Sp Recovery%	Relative Bias%
	Day-1	Apple	0.0953	95%	−5
	Day-2	Spinach	0.0817	82%	−18
	Day-3	Orange	0.119	119%	19
	Day-4	Lettuce	0.111	111%	11
	Day-5	Apple	0.0931	93%	−7
	Day-6	Spinach	0.0708	71%	−29
	Day-7	Orange	0.109	109%	9
	Day-8	Lettuce	0.104	104%	4
	Day-9	Apple	0.0961	96%	−4
	Day-10	Spinach	0.0726	73%	−27
	Day-11	Orange	0.104	104%	4
	Day-12	Lettuce	0.105	105%	5
	n = 12				
	Mean		0.097	−3%	
	SD. P bias (STDEV.P) %			14%	
	SD. Measured Spiked samples (STDEV.S) µg/g		0.015		
	RSD wR %		16%		
	U (bias)			15%	
	Ucom		21%		
	Uexp		42%		

The precision of the method was evaluated by calculating the pooled relative standard deviation (RSD%) across four concentration levels for each representative commodity. The obtained pooled RSDs were 6% for apple, 7% for spinach, 5% for orange, and 4% for lettuce. All values were ≤ 7%, fulfilling the precision requirements of the SANTE Guideline and are therefore considered acceptable.

The reproducibility test of our developed method was carried out according to the SANTE guidelines. Reproducibility was evaluated by analyzing matrix-matched samples spiked with dithianon at one concentration level at 0.1 µg/g for 12 replicates, prepared and analyzed over 12 separate days using the same LC-MS/MS system but with different 4 analysts.

The obtained results were used to calculate the relative standard deviation within-laboratory reproducibility (RSDwR), which represents the variability of the results across days. The mean recoveries at the spiking level ranged between 71% and 119%, and the calculated RSDwR values = 16% and were below the guideline threshold of ≤ 20%, confirming the method’s reproducibility as shown in Table 9 that summarizes the recovery and RSDwR results for the spiked samples.

### Measurement uncertainty

The expanded uncertainty (Uexp) for the determination of dithianon residues was calculated based on the combined uncertainty of the method, which incorporates contributions from both reproducibility and bias, as recommended by the SANTE guidelines.

The final expanded uncertainty for the determination of dithianon was calculated by using last equations from the output data as shown in Table 9 where Uexp = ± 42% which satisfies the SANTE guideline requirement that was not exceeding the 50% default value for Uexp in SANTE.

The validated method demonstrated excellent repeatability, reproducibility and low measurement uncertainty, ensuring its suitability for the determination of dithianon residues in food matrices. All validation parameters complied with the SANTE/11,312/2021 criteria.

As shown in Table 10 comparison between our new analytical method and the previous literature-reviewed methods according to the parameters of the analytical method’s performance has appeared to be high performance and compliance with the SANTE guideline.

**Table 10:** Comparison between our new analytical method and the previous literature-reviewed methods according to analytical method’s performance.

Tested Matrices	Detection Technique	Selectivity	LOQs	Linearity Range	Matrix Effect	Recovery	Precision	Reapeability	Year	References
Frains, Apple and Apple Leaves	Spectrophotometer	No data	0.6 µg/g	2–8 g/L	No data	94.2–98.9%	1%	No data	1999	Balbir C et al.
Water and Fruits	Spectrofluorimeter	No data	10.6 µg/g	0.63–12.5 µg/ml	No data	96–108%	0.76%	No data	2012	Tian et al.
Red Pepper	HPLC-UV	No data	0.03 µg/g	0.03–1.5 µg/ml	No data	72.2–79.1%	≤ 4%	* Pooled RSD% = 4% * 2 validation levels (0.3 and 1.5 µg/g)	2013	Jin Jang et al.
Fruits, Vegetables and Rice	LC-MS/MS	No data	0.1 µg/g	No data	No data	70–120%	≤ 20%	No data	2016	EURL V 2.1.
Water	LC-MS/MS	No data	0.026 µg/L	0.04–4 µg/L	No data	95.3–112.5.3.5%	11.1%	* Pooled RSD% = 4–7% * 3 validation levels (0.1, 0.4 and 2 µg/L)	2016	Alice Passoni et al.
Cherry	HPLC-DAD	No data	0.09 µg/g	0.1–1 µg/ml	Compensation by matrix-matched standards technique	101.23%	0.72%	No data	2018	Sanja LAZIC et al.
Apple Juice and Water	Raman scattering sensing	No data	20 nM	100 nM – 1 µM	No data	97–98.7.7%	No data	No data	2024	Kaiyi Zheng et al.
Fruits and Vegetables	LC-MS/MS	No interferences	0.01–0.05 µg/g	0.001–0.5 µg/ml	Compensation by standard addition technique	85–113%	≤ 8%	* Pooled RSD% = 4–7% * 4 validation levels (0.01, 0.05, 0.1 and 0.5 µg/g) RSDwR% = 16%	2025	Our new method

### Analysis of real samples

To evaluate the practical application and proficiency of our developed method, a preliminary monitoring study was conducted on fifty fresh produce samples (including apple, orange, strawberry, tomato, grape, mango, peach, green beans, potatoes, and lettuce; *n* = 5 per commodity) collected from local markets in Cairo, Egypt in 2025. Samples were prepared and analyzed following our optimized and validated method.

Analysis of the acquired chromatograms confirmed that no dithianon residues were detected (ND) in any of the fifty samples analyzed. This was established by the absence of a peak at the specific retention time of dithianon (± 0.1 min) and the lack of a significant signal exceeding a signal-to-noise ratio (S/N) of 3:1 for the qualifying transition ions.

The absence of detectable dithianon residues in all fifty samples is a notable finding. It is important to note that dithianon is registered in Egypt by the Agricultural Pesticides Committee (APC), primarily for use on grapes, peach peas and mango. Among the commodities included in this survey, only four commodities as grape, peach, peas and mango fall within the registered uses, whereas the other crops analyzed (apple, orange, strawberry, tomato and lettuce) are not registered for dithianon application. This explains the lack of detectable residues in the analyzed samples. This stands in contrast to the European Union, where dithianon is approved under Regulation (EC) No 1107/2009 (with an approval expiry date of 31 January 2027) and has established Maximum Residue Levels (MRLs).

Therefore, the results of this monitoring study align with the crop-specific registration status of dithianon in Egypt. While this dataset offers a high degree of confidence for the sampled commodities, continuous monitoring remains essential to ensure ongoing compliance and to screen for the potential presence of unregistered pesticide applications in the market. Overall, the findings provide clear evidence that Egyptian agricultural products surveyed in this study are completely free from detectable dithianon residues, safe for human consumption, and fully compliant with European Union residue standards. This strongly supports their safety and enhances their marketability for both domestic and international trade. The validated method presented in this work is fit-for-purpose for such surveillance programs.

### Advantages and limitations

Our single-phase extraction method developed in this study offers several clear advantages over conventional protocols, particularly for the determination of dithianon. First, the method is characterized by its simplicity and speed. Unlike traditional QuEChERS approaches, it eliminates the need for partitioning and salting-out steps, thereby reducing the number of manual operations and minimizing potential sources of error such as incomplete phase separation. This streamlining of the procedure shortens the total sample preparation time and enhances reproducibility across multiple analyses.

Second, the approach provides notable cost-effectiveness. By avoiding the requirement for dispersive SPE (d-SPE) kits, which are often expensive and not always necessary for every analyte, the method offers a more economical solution. This makes it particularly attractive for routine monitoring laboratories, where efficiency and cost savings are essential.

Another important advantage lies in the improved stability of the target analyte. Dithianon is well-documented to undergo degradation in aqueous acetonitrile environments. By employing ethyl acetate as the extraction solvent, our method provides a more stable medium that helps preserve the integrity of the analyte. This increased stability likely explains the consistently high recoveries observed during validation.

The method also demonstrates a greener profile. It requires only a moderate solvent volume (10 mL) and makes use of ethyl acetate, which is generally regarded as less toxic and more environmentally benign than acetonitrile. This contributes to reduced laboratory hazards and aligns with the principles of green analytical chemistry^48,49^.

Finally, our method shows excellent analytical performance. Despite its simplicity, it meets all validation requirements outlined in the SANTE guidelines and delivers results comparable to, or in some respects superior to, more complex multi-residue approaches for dithianon determination. The strong performance metrics, as summarized in Table 10, confirm that our method was suitable as a reliable and practical tool for routine DI residue monitoring.

While our single-phase extraction method demonstrates clear advantages for dithianon determination, certain limitations should be acknowledged. Although ethyl acetate offers lower toxicity than acetonitrile, it remains a volatile and flammable solvent, necessitating careful handling and appropriate laboratory safety measures. In addition, the validation was conducted using a selected set of matrices; therefore, the performance of the method in highly complex or fatty commodities (e.g., avocado, nuts, or animal-derived products) may require further testing and possible adaptation. Moreover, while the method provides improved stability for dithianon compared to aqueous acetonitrile systems, long-term stability studies under varying storage and transport conditions were not performed and should be addressed in future work. Finally, given the importance of international trade, there is a need to apply this method to a larger number of imported-bound into Egypt to ensure compliance with global residue regulations and to further confirm the method’s robustness under routine monitoring conditions.

## Conclusion

Our research focused on development and validation of a highly efficient, simple and fast LC ESI (-) MS/MS Q Trap 6500 plus chromatographic method to determine dithianon residues in various fruits and vegetables. The extraction process was optimized by comparing different solvents such as water, acetone, methanol, and ethyl acetate with and without additives. The goal was to establish a simple, quick, cost-effective single phase extraction method that requires minimal amounts of organic solvent. The analytical method was validated based on the criteria outlined in the European SANTE/11,312/2021and Eurachem guidelines for the analytical method validation. The validation parameters met the acceptable standards. The calibration curves exhibited good linearity, and the average recoveries of dithianon in all tested food matrices were within an acceptable range, accompanied by low relative standard deviations. Overall, this developed method proves to be highly valuable and is strongly recommended for the accurate determination of dithianon residues in fruits and vegetables and help in the routine analysis of dithainon in food.

## Data Availability

All data generated or analyzed during this study are included in this manuscript.
